# Knowledge of Obstructive Sleep Apnea Among the General Population in Arar, Northern Region of Saudi Arabia

**DOI:** 10.7759/cureus.51529

**Published:** 2024-01-02

**Authors:** Ghazala Rasool, Wajd H Alenezi, Rahaf Muteb S Alanazi, Hala A Almadai, Norah N Alsharif

**Affiliations:** 1 Internal Medicine, Northern Border University, Arar, SAU; 2 General Practice, The Northern Borders Health Cluster, Arar, SAU; 3 General Practice, Alamal Complex for Mental Health, Arar, SAU; 4 General Medicine and Surgery, Northern Border University, Arar, SAU

**Keywords:** sleep disordered breathing (sdb), ksa, awareness, obstructive sleep apnea, osa

## Abstract

Background: Obstructive sleep apnea (OSA) can have serious health consequences if left untreated, including an increased risk of heart disease, stroke, and diabetes. Raising awareness about this condition can help reduce the stigma surrounding sleep disorders and encourage individuals to seek help if they suspect they may have OSA. It is important for the general population to be informed about this condition in order to promote better overall health and well-being.

Objective: To assess the level of knowledge of OSA among the general population of Arar, Northern region of Saudi Arabia.

Methodology: This study is a cross-sectional descriptive study to assess the knowledge of OSA in the general population in the northern region of Saudi Arabia. We used an online self-administered questionnaire to collect sociodemographic data and questions about the subject’s awareness.

Results: Four hundred thirty-nine individuals participated in this study. The majority of them 337 (76.8%) were female. About 181 (41.2%) of the studied participants were in the age category of 15-25 years and 66.3% had heard about sleep apnea. Nearly 316 (72%) reported that sleep apnea is considered dangerous. Snoring, low sleep quality, and coughing were the main symptoms of sleep apnea. Moreover, obesity, smoking, and chronic diseases were the main causes of sleep apnea, according to the participants' knowledge. About 37 (8.4%) of participants said that they were diagnosed with sleep apnea, and 30 (6.8%) of them revealed that sleep apnea affected their quality of life.

Conclusion: Knowledge of OSA in Saudi Arabia is considered inadequate. General population knowledge of OSA can be improved through a multi-faceted approach that involves public education campaigns, continuous medical education for healthcare professionals, and policy-level interventions.

## Introduction

Obstructive sleep apnea (OSA) is a prevalent medical problem that falls under the category of sleep-disordered breathing (SDB). It is notable by frequent incidents of full or partial upper airway failure. According to the frequency of apneas and/or hypopneas per hour of sleep, the index of apnea-hypopnea (AHI) is employed to define the disorder as severe, moderate, or mild. Polysomnography (PSG) or other sleeping monitoring methods can be used to diagnose OSA [1].

The condition known as OSA was initially recognized in 1965. For a significant period of time, medical professionals viewed OSA as a straightforward and sporadic blockage of the upper airway, leading to early treatments that centered on addressing this airway obstruction. Prior to 1980, the sole successful treatment for OSA was tracheostomy, a procedure that involved bypassing the obstruction in the upper airway [2].

The etiology of OSA is multifactorial, involving elements such as obesity, distribution of body fat in the center, circumference of the neck, and anomalies of the craniofacial anatomy, all contributing to the development of the condition with variations among individuals. OSA patients experience recurrent narrowing or blockage of the pharyngeal airway during sleep [3]. Pathophysiologically, several mechanisms have been implicated, including anatomical compromise, dysfunction of pharyngeal dilator muscles, lowered threshold for arousal, and instability in tethering of diminished lung volume and/or ventilatory control, all contributing to the manifestation of OSA [4]. Furthermore, obesity plays a significant role in OSA, as demonstrated by the reduction in OSA severity following weight loss interventions, coinciding with the increased prevalence of OSA alongside rising obesity rates. While OSA is most frequently observed in older males, it can also affect women and children [5].

OSA must be considered whenever a patient exhibits extreme daytime sleepiness, snoring, and choking or gasping during sleep, especially if risk factors are present. Other signs include headaches in the morning and concomitant illnesses such as hypertension [6]. Although detailed inquiries to investigate the many reasons for daytime sleepiness, snoring, and neuropsychiatric disorder may aid in distinguishing OSA from other conditions, sleep apnea testing is essential to make a determination of OSA [6].

The most prevalent sleep-related respiratory condition is OSA. In the Wisconsin Cohort Study, Young et al. evaluated the frequency of middle-aged people with OSA, working individuals and exposed that severe to moderate OSA developed at rates that varied from 3.7% to 4.4% in women. Men had rates that were between two and three times higher, ranging from 6.2% to 11% [7].

In Saudi Arabia, the prevalence of OSA is high in four out of 10 middle-aged Saudi women and three out of 10 middle-aged Saudi males [3]. Furthermore, OSA with an apnea-hypopnea index higher than 10/hour was present in 56% of Saudi patients with acute coronary syndrome who were hospitalized in the critical care unit [3].

The prevalence of OSA also varies by race. African Americans under the age of 35 years old have a higher prevalence rate compared with Caucasians of the same age group, regardless of body weight. The prevalence of OSA in Asia has a similar rate to that in the United States, despite lower obesity rates [6].

It is important for patients with OSA to consider the various treatment options available, including lifestyle changes and positive pressure treatments. These options can greatly improve their quality of life and overall health. Continuous-positive airway-pressure (CPAP) is the first line of treatment for OSA since it is so effective. Also, research on CPAP has shown that it lowers blood pressure, the risk of stroke, and the incidence of dysrhythmias in addition to enhancing quality of life and sleep. Oral appliance therapy is an option for patients who are unable to tolerate CPAP. The two types of oral appliance therapies are tongue-retaining devices and mandibular advancement devices, which are the preferable option. Moreover, a variety of surgeries, including laryngeal, nasal, oral, and hypopharyngeal operations, are available to address any anatomical anomalies producing obstruction. Obese patients can benefit from bariatric operations [8].

The issue of OSA is a significant concern for individuals who suffer from this chronic morbid condition. It is unfortunate that many patients are unaware of the extent to which this disease can impact their quality of life. Furthermore, there is a dearth of studies that have assessed the level of awareness and knowledge of OSA among the general population in Saudi Arabia. Therefore, the research being conducted to determine adults' awareness and understanding of OSA in the Kingdom of Saudi Arabia's northern area is of utmost importance. This study aims to provide valuable insights to researchers and healthcare professionals, and ultimately benefit individuals who are affected by OSA. It is crucial that we continue to prioritize research in this area to improve the understanding and management of this condition.

## Materials and methods

Study type and duration

This is a cross-sectional descriptive study that offers insightful information about the general population of Saudi Arabia's northern area, Arar during the year 2023.

Study population

The research guarantees a heterogeneous representation of persons above the age of 15, comprising both Saudi and non-Saudi residents of the area. The study's emphasis on the particular demographic of interest is further strengthened by excluding individuals who were younger than fifteen or who came from a different portion of Saudi Arabia.

Data collection tool and method

An anonymous online survey instrument was used for the study, and it was distributed to Saudi individuals living in the northern region of the country. Following the reading and acceptance of the informed consent, it was created as an Arabic-language, self-administered online questionnaire. This survey asks questions regarding sociodemographic information as well as issues pertaining to sleep.

Sampling

A straightforward random sample method was used to get the data.

Data management and analysis

The data was input into SPSS and stored without any attempt to identify the participants because the questionnaire does not contain any personal information that may be used to identify a participant, such as a name, ID number, or other specific information. With SPSS (version 26), data analysis was carried out. The relationship was computed using the Chi-Square test.

The numerical numbers (mean, frequency, etc.) and percentages representing the qualitative aspects were shown in the figures. The significance threshold for each test used in the investigation was set at 0.05. The clearance was given by the Ethics Committee of Northern Border University College of Medicine (Approval number: 7/44/H). This research offers a thorough and in-depth examination of the population's sleep-related and demographic data in Arar, a city in northern Saudi Arabia.

## Results

Table 1 shows the sociodemographic characteristics of the participants. About 439 individuals participated in this study. The majority of them (76.8%, n=337) were female. About 41.2 % (n=181) of the studied participants were in the age category of 15-25 years. The majority of them were from urban areas (95% n=417). As regards marital status, more than half of them were married (52.4%, n=230). About 35.5% (n=156) of them were working in the health sector.

**Table 1:** Sociodemographic characteristics of the participants (N=439)

Variable		No	%
Age	15-25	181	41.2%
	26-35	78	17.8%
	36-45	100	22.8%
	>45 years	80	18.2%
Gender	Male	102	23.2%
	Female	337	76.8%
Education level	Basic education	4	0.9%
	High school	87	19.8
	University or more	348	79.3%
Residence	Urban	417	95%
	Rural	22	5%
Social status	Single	209	47.6%
	Married	230	52.4%
Occupation	Student	153	34.9%
	Unemployed	89	20.3%
	Officer	171	39.0%
	Retired	26	5.9%
Work in health sector	Yes	156	35.5%
	No	283	64.5%

Table 2 shows sleep characteristics among participants. Nearly half of the participants reported that their sleeping hours per day were 7-9 hours. The majority of them (69%, n=303) said that sleep hours were enough to perform daily tasks without hindrances. However, 62.9% (n=276) suffered from drowsiness during the day. About 61.5% (n=270) felt they needed to take a nap during the day.

**Table 2:** Distribution of participants according to sleep characteristics (N=439)

Variable	Answer	No (%)
1) Your sleeping hours per day	3 hours or less	5 (1.1%)
	4-6 hours	180 (41.0%)
	7-9 hours	218 (49.7%)
	More than 9 hours	36 (8.2%)
2) Sleep hours are enough to perform daily tasks without hindrances	Yes	303 (69%)
	No	136 (31%)
3) Do you suffer from drowsiness during the day?	Yes	276 (62.9%)
	No	163 (37.1%)
4) Do you need to take a nap during the day?	Yes	270 (61.5%)
	No	169 (38.5%)

Table 3 shows the participant's knowledge of sleep. Most of them (84.7%, n=372) agreed that a person needs 7-9 hours of sleep per day. Additionally, more than half of them (54.2%, n=238) reported that the most important benefit of adequate sleep was mental health. Moreover, most of them (97.5%, n=428) agreed that a lack of sleep affects work. According to their opinion, sports activities were the main factor that improved sleep, and psychological stressors were the main factor that might disturb sleep.

**Table 3:** Distribution of participants according to their knowledge of sleep (N=439)

Variable	Answer	No (%)
Number of sleeping hours a person needs in a day	3 hours or less	1 (0.2%)
	4-6 hours	27 (6.2%)
	7-9 hours	372 (84.7%)
	More than 9 hours	39 (8.9%)
The most important benefit of adequate sleep	Mental benefits	238 (54.2%)
	Physical benefits	139 (31.7%)
	Social benefits	4 (0.9%)
	Psychological benefits	58 (13.2%)
Lack of sleep affects work	Yes	428 (97.5%)
	No	11 (2.5%)
Napping during the day affects sleep in the evening	Yes	286 (65.1%)
	No	153 (34.9%)
Which of the following may improve sleep?	Phone use	28 (6.4%)
	Watching TV	36 (8.2%)
	Use of medicines	36 (8.2%)
	Sports activities	193 (44%)
	Lack of food	35 (8%)
	Eating a lot	66 (15%)
	Don’t know	45 (10.3%)
Which of the following might disturb sleep?	Sports activities	6 (1.4%)
	Eating a lot	27 (6.2%)
	Psychological stressors	173 (39.4%)
	Drink coffee and tea	105 (23.9%)
	Pain and health problems	121 (27.5%)
	I do not know	7 (1.6%)

About 66.3% (n=291) heard about sleep apnea. Nearly 72% (n=316) reported that sleep apnea is considered dangerous. Snoring, low sleep quality, and coughing were the main symptoms of sleep apnea. Moreover, obesity, smoking, and chronic diseases were the main causes of sleep apnea, according to the participants' knowledge. Social media was the main source of information about sleep apnea (Table 4).

**Table 4:** Distribution of participants according to their knowledge of sleep apnea (N=439)

Variable	Answer	No (%)
Heard of sleep apnea	Yes	291 (66.3%)
	No	148 (33.7%)
Sleep apnea is considered dangerous	Yes	316 (72%)
	No	14 (3.2%)
	Don't know	109 (24.8%)
Symptoms of sleep apnea	Snoring	141 (32.1%)
	Paralysis	9 (2.1%)
	Sweating	76 (17.3%)
	Low sleep quality	112 (25.5%)
	Choking	101 (23%)
Causes of sleep apnea	Congenital defects in the respiratory tract	33 (7.5%)
	Smoking	103 (23.5%)
	Facial injuries	28 (6.4%)
	Obesity	157 (35.8%)
	Chronic diseases	94 (21.4%)
	Don’t know	24 (5.5%)
Do you know methods of diagnosing sleep apnea?	Yes	88 (20%)
	No	351 (80%)
Do you know management of sleep apnea?	Yes	95 (21.6%)
	No	344 (78.4%)
Diagnosis and treatment of sleep apnea is important	Yes	349 (79.5%)
	No	13 (3%)
	May be	77 (17.5%)
Source of information about sleep apnea	Relatives and friends	15 (3.4%)
	The doctor	110 (25.1%)
	Social media	129 (29.4%)
	The media	63 (14.4%)
	Not interested	95 (21.6%)
	Other	27 (6.2%)

**Figure 1:**
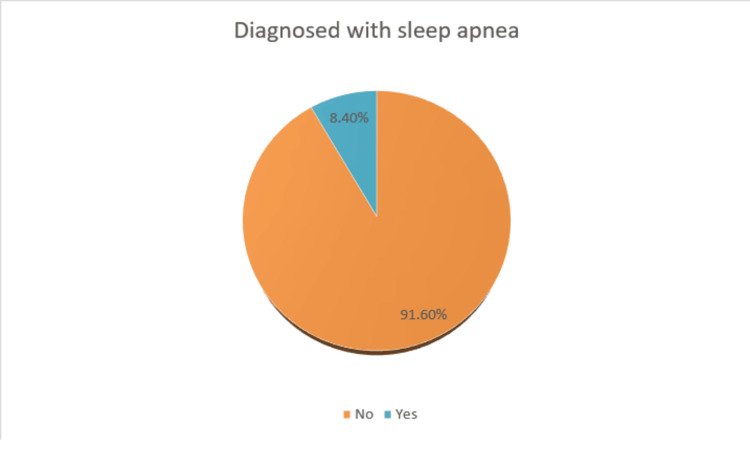
Prevalence of sleep apnea among study participants

As shown in Figure 1, about 8.4% of participants said that they were diagnosed with sleep apnea.

Table 5 shows the relationship between knowledge of sleep and gender. Among participants, 39.9% (n=175) of females reported that mental benefits were the most important benefits of adequate sleep, compared to 14.4% (n=63) of males who reported the same opinion. Additionally, nearly three-quarters of females agreed that work was affected by lack of sleep, compared to 22.3% (n=98) of males. A statistically significant difference is found between females and males as to their knowledge about whether napping during the day affects sleep in the evening. About 47.2% (n= 207) and 12.3% (n= 54) of females and males, respectively, established that there was a relationship between sleep and aging.

**Table 5:** Relation between knowledge of sleep and gender among participants (N=439) *Chi-Squared Tests

Variable	Female (N=337) (78.6%)	Male (N=102) (23.2%)	P-value*
	No (%)	No (%)	
The most important benefit of adequate sleep			
Mental benefits	175 (39.9%)	63 (14.4%)	0.057
Physical benefits	117 (26.7%)	22 (5%)	
Social benefits	2 (0.5%)	2 (0.5%)	
Psychological	43 (9.8%)	15 (3.4%)	
Lack of sleep affects work			
Yes	330 (75.2%)	98 (22.3%)	0.296
No	7 (1.6%)	4 (0.9%)	
Napping during the day affects sleep in the evening			
Yes	208 (47.4%)	78 (17.8%)	0.006
No	129 (29.4%)	24 (5.5%)	
There is a relationship between sleep and aging			
Yes	207 (47.2%)	54 (12.3%)	0.243
No	42 (9.6%)	13 (3%)	
Don’t know	88 (20%)	35 (8%)	

Table 6 shows the relationship between awareness of sleep apnea and gender. About 63.6% (n= 279) of females and 16.4% (n= 72) of males reported that they did not know methods of diagnosing sleep apnea. And 63.6% (n= 279) of females and 15.9% (n= 70) of males agreed that the diagnosis and treatment of sleep apnea were important. The percentage of females who said that they were diagnosed with sleep apnea was higher than that of males, with 4.6% (n=20) among females and 3.9% (n=17) among males. These associations are statistically significant.

**Table 6:** Relation between awareness of sleep apnea and gender among participants (N=439) *Chi-Squared Tests

Variable	Female (N=337) (78.6%)	Male (N=102) (23.2%)	P-value*
	No (%)	No (%)	
Heard of sleep apnea			
Yes	217 (49.4%)	74 (16.9%)	0.127
No	120 (27.3%)	28 (6.4%)	
Sleep apnea is considered dangerous			
Yes	243 (55.4%)	73 (16.6%)	0.518
No	9 (2.1%)	5 (1.1%)	
Don’t know	85 (19.4%)	24 (5.5%)	
Do you know methods of diagnosing sleep apnea?			
Yes	58 (13.2%)	30 (6.8%)	0.007
No	279 (63.6%)	72 (16.4%)	
Do you know management of sleep apnea?			
Yes	59 (13.4%)	36 (8.2%)	0.001
No	278 (63.3%)	66 (15%)	
Diagnosis and treatment of sleep apnea is important			
Yes	279 (63.6%)	70 (15.9%)	0.008
No	8 (1.8%)	5 (1.1%)	
May be	50 (11.4%)	27 (6.2%)	
Are you diagnosed with sleep apnea?			
Yes	20 (4.6%)	17 (3.9%)	0.001
No	317 (72.2%)	85 (19.4%)	

## Discussion

OSA is a common sleep disorder characterized by repeated episodes of partial or complete obstruction of the upper airway during sleep, leading to disrupted breathing patterns and inadequate oxygen supply to the body. It is a serious condition that can have significant health implications if left untreated [2,9]. When it comes to the knowledge of OSA in Saudi Arabia, it is essential to recognize the current state of understanding among the general population, healthcare professionals, and policymakers. By doing so, we can identify areas that need improvement and develop strategies to enhance awareness and knowledge about this sleep disorder. One key aspect to consider is the prevalence of OSA in Saudi Arabia. While there is limited data available specifically for Saudi Arabia, studies from neighboring countries and the global prevalence rates suggest that OSA is a significant health issue in the region. It is estimated that around 9-10% of the general population worldwide suffers from OSA, with higher rates observed in certain populations such as obese individuals, older adults, and those with certain medical conditions [5].

According to our study results, 291 (66.3%) heard about sleep apnea. Snoring, low sleep quality, and coughing were the main symptoms of sleep apnea. Moreover, obesity, smoking, and chronic diseases were the main causes of sleep apnea, according to the participants' knowledge. A study evaluating the general public's knowledge and awareness of OSA in the Asir region of Saudi Arabia was published in 2019, and the findings revealed a low degree of awareness about all elements of OSA [9]. The general public was not well-informed about the problems of OSA, despite a promising outcome from a different study conducted in France [10]. In a 2017 study carried out in Singapore, it was shown that while 21.5% of individuals knew what OSA was, only 13% could characterize it accurately [11]. Dental interns in Saudi Arabia demonstrated a low level of understanding of OSA [12], while medical students appeared to know very little about the condition during their clinical semesters [13-15]. This was greater than what was found in a study from Saudi Arabia, where it was shown that almost two-thirds of participants (60.4%) had heard of sleep apnea and that the sample (72.4%) believed it to be dangerous. Only 14.6% of cases are aware of the procedures for diagnosing sleep apnea, and only 11.6% are aware of the choices for managing the condition [16]. However, the majority, or 75.6%, agreed that sleep apnea diagnosis and treatment are crucial. A study that included 1,000 members of all Saudi citizens found that the sample's overall knowledge scores ranged from bad for 80.7% to good for 19.3%, with a mean of 24.8%; treatment techniques knowledge scores were 26%, risk factors were 24.5%, and complications were 20.9% on average [17]. Sixty-four percent of participants in a different survey with 626 people in the Asir region of Saudi Arabia were aware of OSA, compared to only 36% who were not. Almost all respondents believed that OSA was hazardous; 24% were unsure; and 81% were uninformed of the processes for diagnosing OSA. While 84% of respondents did not know how to cure OSA, 80% of them agreed that diagnosing and treating sleep apnea is essential [9].

Another study involving 352 dental professionals was carried out in Jeddah; the majority of respondents (80.6%) indicated that they had prior knowledge of OSA in the self-assessment question. However, the average overall knowledge score of 9.86 fell short of the 12-point criteria, and 65.58% of participants had a score below 60%, indicating that they had little knowledge of OSA [18]. Only 13.0% of respondents correctly defined OSA in a population-based study conducted among 1306 participants in Singapore, and just 5.9%, 12.1%, 11.5%, and 8.4% of participants accurately identified at least one OSA symptom, health consequence, and available therapy, respectively. This shows that there is currently a poor level of OSA awareness and knowledge among the general public.

A cross-sectional analytical study was undertaken in Turkey with 1651 patients and patient families who applied to the outpatient clinics at Konya Research and Training Hospital. Only 39% of the participants felt they knew enough about OSAS, even though 61% had never heard of it. Individuals with little, medium, and strong knowledge made up, respectively, 37.3%, 54.3%, and 8.4% of the total [19]. There is a poor level of knowledge and reporting of OSA symptoms, according to a different study conducted in Nigeria [20]. The severity of the risk factors and symptoms of sleep apnea were generally understood by Canadians [21]. In a research conducted in China, 21.5% of the respondents were informed about OSA. A total of 77 (5.9%), 158 (12.1%), 150 (11.5%), and 110 (8.4%) respondents, respectively, were able to accurately state at least one risk factor, symptom, health consequence, and treatment option for OSA [15].

It's interesting to note that while the majority of people feel they are getting enough hours of sleep, a significant number still feel the need to take a nap during the day. This could be due to various factors such as poor sleep quality, high levels of stress, or simply not getting enough restful sleep during the night. It's important to address these issues and prioritize getting enough quality sleep to avoid the need for naps during the day, as consistent napping could be a sign of underlying sleep deprivation.

According to our results, there was no statistical association between the gender of participants and their knowledge of OSA. There was no discernible difference between male and female OSA knowledge, according to research by Sia et al. [10]. In contrast, a different study found that men and women have different levels of general knowledge, with a significance difference of p = 0.007. In terms of knowledge, the 50 to 59-year-old age group had the highest levels [17]; however, in France, people under the age of 40 had a greater understanding of the symptoms and problems [11].

In terms of awareness among the general population, it is crucial to assess the level of understanding about OSA, its symptoms, risk factors, and available treatment options. Public education campaigns and initiatives can play a vital role in disseminating accurate information about OSA, its impact on overall health, and the importance of seeking medical attention. These campaigns can utilize various channels such as social media, television, radio, and community outreach programs to reach a wide audience. Another key aspect is the knowledge and training of healthcare professionals in Saudi Arabia. It is essential for healthcare providers to be well-informed about OSA, its diagnosis, and treatment options to effectively identify and manage patients with this condition. Continuous medical education programs can be implemented to update healthcare professionals about the latest advancements in the field of sleep medicine, including the diagnosis and management of OSA. This can help improve the quality of care provided to individuals suffering from OSA and ensure that they receive appropriate treatment.

Additionally, it is important to involve policymakers and healthcare authorities in efforts to raise awareness and knowledge about OSA. By recognizing OSA as a significant public health issue, policymakers can allocate resources and develop policies that prioritize the prevention, diagnosis, and treatment of this sleep disorder. This can include funding research studies, establishing specialized sleep centers, and incorporating sleep health education into the curriculum of medical schools and residency programs.

Study limitations

The study provided valuable insights into the awareness and understanding of OSA in the region. However, it is important to acknowledge the limitations of the study, such as the potential for selection bias in the survey sample due to the large proportion of females, the reliance on self-reported data which may be subject to recall bias, and the lack of detailed information on the demographic characteristics of the participants. Also, data was collected by distributing questionnaires through social media platforms which may cause bias. Additionally, the study's findings may not be generalizable to other regions of Saudi Arabia or to different cultural or socioeconomic groups. Despite these limitations, the study still contributes to our understanding of OSA awareness in the region and highlights the need for further research and education on this important health issue.

## Conclusions

The knowledge of OSA in Saudi Arabia is inadequate. The knowledge of OSA can be improved through a multi-faceted approach that involves public education campaigns, continuous medical education for healthcare professionals, and policy-level interventions. By enhancing our understanding of OSA, its prevalence, and the importance of early diagnosis and treatment, we can strive toward better sleep health and overall well-being in the population of Saudi Arabia.

It is crucial to explore the gap in information regarding OSA in order to assist policymakers in designing effective health education sessions. By identifying the areas where knowledge is lacking, we can ensure that the information provided to the public is comprehensive and accurate. Additionally, having data that covers the gap in knowledge will help in addressing the specific needs of individuals affected by OSA. This will ultimately contribute to better health outcomes and improved awareness of this condition.
